# Distribution patterns of microsatellites and development of its marker in different genomic regions of forest musk deer genome based on high throughput sequencing

**DOI:** 10.18632/aging.102895

**Published:** 2020-03-10

**Authors:** Wen-Hua Qi, Ting Lu, Cheng-Li Zheng, Xue-Mei Jiang, Hang Jie, Xiu-Yue Zhang, Bi-Song Yue, Gui-Jun Zhao

**Affiliations:** 1 Chongqing Engineering Laboratory of Green Planting and Deep Processing of Three Gorges Reservoir Famous-region Drug, College of Biology and Food Engineering, Chongqing Three Gorges University, Chongqing 404120, P. R. China; 2 Key Laboratory of Bio-resources and Eco-environment (Ministry of Education), College of Life Sciences, Sichuan University, Chengdu 610064, P. R. China; 3 Sichuan Institute of Musk Deer Breeding, Chengdu 611830, P. R. China; 4 College of Environmental and Chemistry Engineering, Chongqing Three Gorges University, Chongqing 404120, P. R. China; 5 Chongqing Engineering Technology Research Center for GAP of Genuine Medicinal Materials, Chongqing Institute of Medicinal Plant Cultivation, Chongqing 408435, P. R. China

**Keywords:** microsatellites, GC, variation analysis, genomic regions, forest musk deer genome

## Abstract

Forest musk deer (*Moschus berezovskii*, FMD) is an endangered artiodactyl species, male FMD produce musk. We have sequenced the whole genome of FMD, completed the genomic assembly and annotation, and performed bioinformatic analyses. Our results showed that microsatellites (SSRs) displayed nonrandomly distribution in genomic regions, and SSR abundances were much higher in the intronic and intergenic regions compared to other genomic regions. Tri- and hexanucleotide perfect (P) SSRs predominated in coding regions (CDSs), whereas, tetra- and pentanucleotide P-SSRs were less abundant. Trifold P-SSRs had more GC-contents in the 5′-untranslated regions (5'UTRs) and CDSs than other genomic regions, whereas mononucleotide P-SSRs had the least GC-contents. The repeat copy numbers (RCN) of the same mono- to hexanucleotide P-SSRs had different distributions in different genomic regions. The RCN of trinucleotide P-SSRs had increased significantly in the CDSs compared to the transposable elements (TEs), intronic and intergenic regions. The analysis of coefficient of variability (CV) of P-SSRs showed that the RCN of mononucleotide P-SSRs had relative higher variation in different genomic regions, followed by the CV pattern of RCN: dinucleotide P-SSRs > trinucleotide P-SSRs > tetranucleotide P-SSRs > pentanucleotide P-SSRs > hexanucleotide P-SSRs. The CV variations of RCN of the same mono- to hexanucleotide P-SSRs were relative higher in the intron and intergenic regions, followed by that in the TEs, and the relative lower was in the 5'UTR, CDSs and 3'UTRs. 58 novel polymorphic SSR loci were detected based on genotyping DNA from 36 captive FMD and 22 SSR markers finally showed polymorphism, stability, and repetition.

## INTRODUCTION

Forest musk deer (*Moschus berezovskii*, FMD) is an endangered artiodactylous species [1], which has been listed on the IUCN Red List of Threatened Species and the Appendices of the CITES. In addition, the FMD is also included as the first grade protected species under the Wild Animal Protection Law in China since 2002 [2]. Males and female FMDs have no antlers, and their hind legs are about 1/4 longer than the forelegs, indicating a tendency to move by leaping. Male FMD has long, upper canine teeth that project downward up to 5 cm below the lips that are used for threat and self-display. The most unique characteristic is the musk sac or pod which the males possess [2, 3]. For a long time, musk deer has been valued for their musk in the musk sac, secreted by the musk gland possessed by the males. The musk liquid, a jelly-like milky white substance, is preliminarily secreted and becomes a powdery and ripe musk with strong odor and brown color when it develops and dries gradually. Wild musk deers are killed to cut off the musk sac from the mature males between the genital organs and the umbilicus, as they made a profit in the trade.

Microsatellites (or simple sequence repeats, SSRs) are tandem repetitions of 1–6 bp oligonucleotide repeat units of DNA sequences [4], which are widely distributed in both coding and noncoding regions of eukaryotic and prokaryotic genomes [5, 6]. SSRs have owned significant features of high reproducibility, high polymorphism, selective neutrality, codominant inheritance, abundance and genome-wide coverage [7], which have been widely employed in population genetics, phylogenetics, genetic mapping, linkage and kinship relationships [8]. SSRs are involved in chromatin fractions, gene expression and regulation, and transcription and protein function. The traditional methods of developing SSR markers are screening genomic DNA libraries or constructing SSR-enriched libraries, both of which are often costly, time-consuming, and labor-concentrating [9, 10]. The cost of sequencing is reduced with the fast development of high-throughput sequencing technology. Analysis technology of bioinformatics is also developing rapidly, the analysis results of which are more reliable. Thus, we have sequenced the whole genome of FMD, completed the genome assembly and annotation process. However, basing on the obtainability of genome sequences, it makes possible to mine SSRs in a large scale as genome level, and SSRs analysis is helpful to explore their distributions, functions, and evolution [11, 12].

The number of captive FMD populations is small, and the gene communication of different captive populations is difficult. These facts lead to inbreeding in different captive FMD populations. Therefore, it is very necessary and urgent to develop high quality genetic markers and carry out genetic diversity and genetic management of FMD. All of the existing SSR markers of FMD were dinucleotide repeats [13]. Compared with dinucleotide repeats, tetranucleotide SSR makers can gain more accurate and reliable genotyping. In the FMD genome, we detected and characterized SSRs and examined their distributions and variations in different genomic regions, including their occurrences in 5'-untranslated regions (5′UTRs), protein-coding regions (CDSs), introns, 3′UTRs and intergenic regions. SSR motifs were mined and characterized in the different genomic regions of FMD genome. Furthermore, GC-content of SSRs was analyzed in the FMD genome. The study may help to explore the variation analysis of repeat copy numbers of SSRs in the different genomic regions of FMD genome. Finally, we have used these SSRs sequences for developing polymorphic SSR markers for population genetics studies.

## RESULTS

### The number, relative abundance and proportion of mono- to hexanucleotide P-SSRs in FMD genome

A total of 680,635 perfect (P) SSRs were identified in the FMD genome, the relative abundance of which was 247.75#/Mb (Table 1). Mononucleotide P-SSRs were the most abundant category, accounting for 40.19% of all SSRs, next was the pattern: di- > tri- > penta- > tetra- > hexanucleotide P-SSRs. In comparison, tetra- and hexa-nucleotide P-SSRs were less abundant. The most GC-content was in the tri- and hexanucleotide P-SSRs, and the least was in the mononucleotide P-SSRs. In comparison, the GC-content in mono- and tetranucleotide P-SSRs was less than that in genome-wide level, and the GC-content in the rest P-SSRs was more than that in genome-wide level.

**Table 1:** Overview of mono- to hexanucleotide P-SSRs in the FMD genome.

**Type**	**Mono-**	**Di-**	**Tri-**	**Tetra-**	**Penta-**	**Hexa-**	**Total**
# of P-SSRs	273,518	148,175	122,105	399,77	962,62	598	680,635
GC-content (in %)	1.72	37.14	62.56	29.13	40.27	59.97	32.41
Total length of P-SSRs (bp)	3,192,531	2,719,558	2,077,536	678,452	106,801	15,420	8,790,299
Relative abundance (#/Mb)	100.38	53.40	44.00	14.41	35.33	0.22	247.75
P-SSR percentage (%^a^)	40.19	21.77	17.94	5.87	14.14	0.09	100.00

%^a^= mono- to hexanucleotide P-SSRs account for the proportion of all P-SSRs in the whole FMD genome.

The most frequent P-SSR motifs for different length were counted in the FMD whole genome level (Table 2). In the mononucleotide repeat type, the unit (A)_n_ were predominant (> 80 #/Mb), while (C)_n_ repeats were rare (< 2 #/Mb). (AC)_n_, (AT)_n_, and (AG)_n_ were the three most common dinucleotide SSRs units, the three of which accounted for over 99% of all dinucleotide SSRs. In comparison, the (AC)_n_ was particularly dominant, and (CG)_n_ was the least common unit. Among the trinucleotide repeat type, (ACG)_n_ and (AGC)_n_ were the most common repeat units, next were the (AAC)_n_, (AAT)_n_, (ACC)_n_ and (CCG)_n_, and the (ACT)_n_ motif was the least common in the FMD genome. In the tetranucleotide P-SSRs, the (AAAT)_n_ unit was the most common motifs, the next were the (AAAC)_n_ and (AAAG)_n_, and the (CCGG)_n_ motif was the least common in the FMD genome. Penta- and hexanucleotide P-SSRs have a great deal of motifs in the FMD genome. The (AACTG)_n_ and (AGTTC)_n_ were the two most common pentanucleotide motifs, the (AACCCT)_n_, (ACCCCC)_n_, and (AGGGTT)_n_ motif were most frequent hexanucleotide motifs.

**Table 2:** The most frequent P-SSR motifs in the FMD genome.

**Repeat motifs^a^**					
**Mono-**	**Di-**	**Tri-**	**Tetra-**	**Penta-**	**Hexa-**
A(80.48)	AC (34.47)	ACG (17.94)	AAAT (4.12)	AACTG (0.87)	AACCCT (0.02)
C(1.08)	AG (4.17)	AGC (17.74)	AAAC (1.78)	AGTTC (0.87)	ACCCCC (0.02)
—	AT (14.62)	AAC (2.00)	AAAG (0.88)	AAGTG (0.02)	AGGGTT (0.02)
—	CG (0.14)	AAT (1.97)	AGGT (0.64)	AAACA (0.01)	AAACAA (0.01)
—	—	ACC (1.39)	ACGT (0.61)	AAGGC (0.01)	ACACAG (0.01)
—	—	CCG (1.09)	ACCT (0.59)	GCCTT (0.01)	ACTGCT (0.01)

^a^ The numbers in parentheses refer to relative abundance.

### Difference of P-SSRs in different genomic regions of FMD genome

**Figure 1:**
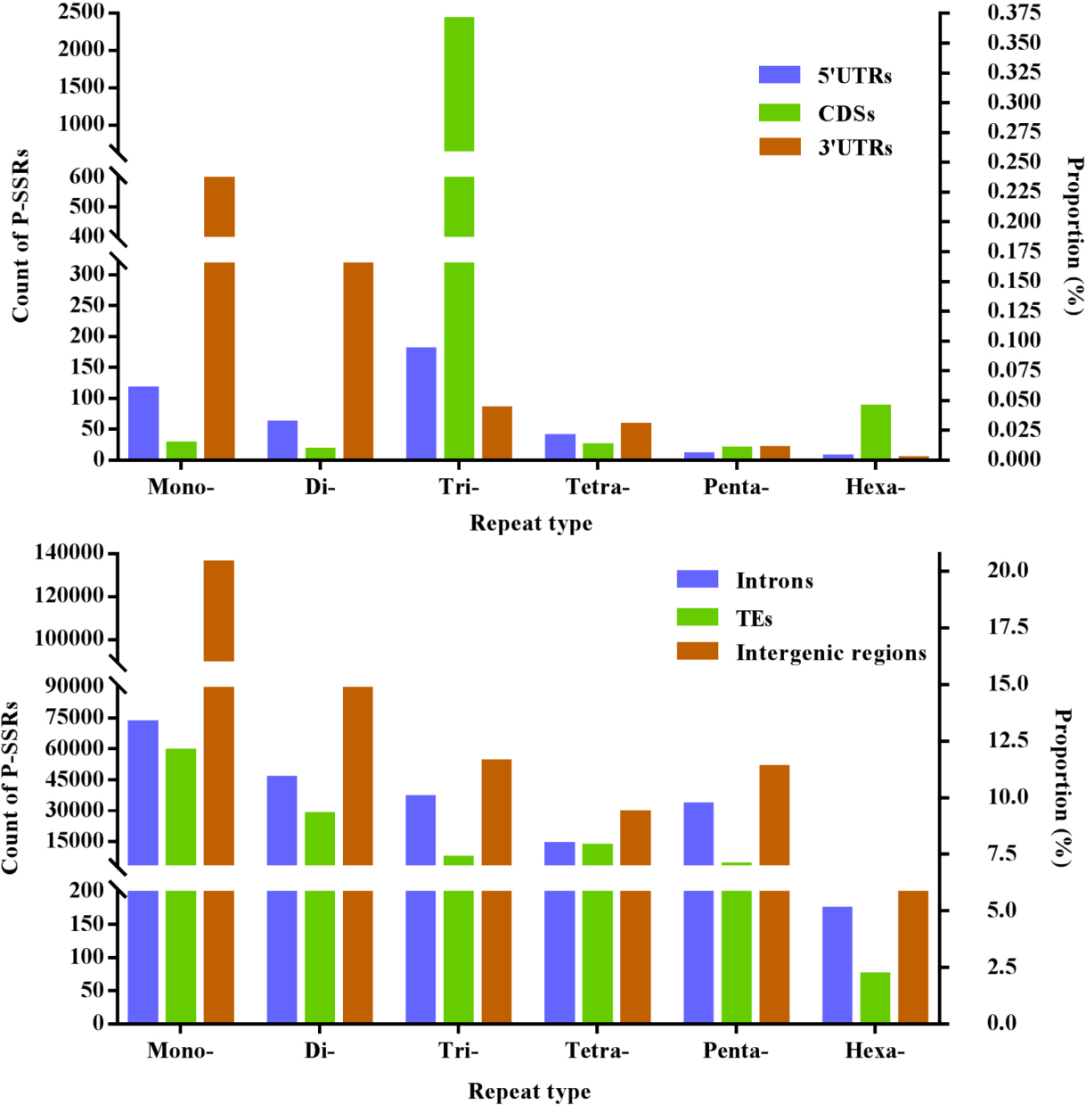
**The proportion of mono- to hexanucleotide P-SSRs in different genomic regions of FMD genome.**

Difference of mono- to hexanucleotide P-SSRs was analyzed in different genomic regions of FMD genome, and these results were indicated in Figure 1 and Table 3. In the 5′UTR regions, trinucleotide P-SSRs was the most abundant type, next was the pattern: mono- > di- > tetra- > penta- > hexa-nucleotide P-SSRs. In the CDSs, trinucleotide P-SSRs was the most abundant type, next was the pattern: hexa- > mono- > tetra- > penta- > dinucleotide P-SSRs. In the introns, mononucleotide P-SSRs was the most abundant type, next was the pattern: di- > tri- > penta- > tetra- > hexanucleotide P-SSRs. In the 3′UTRs, mononucleotide P-SSRs was the most abundant type, next was the pattern: di- > tri- > tetra- > penta- > hexanucleotide P-SSRs. In the TEs, mononucleotide P-SSRs was the most abundant type, next was the pattern: di- > tetra- > tri- > penta- > hexanucleotide P-SSRs. In the intergenic regions, mononucleotide P-SSRs was the most abundant type, next was the pattern: di- > tri- > penta- > tetra- > hexanucleotide P-SSRs. In comparison, the intronic and intergenic regions had the most total P-SSR abundance comparing with other genomic regions in the FMD genome (Table 3). Therefore, it was inferred that P-SSRs were less abundant in protein-coding regions than non-coding regions in the FMD genome.

**Table 3:** Number, percentage, and relative abundance of P-SSRs in the different genomic regions of the FMD genome.

**Repeat type**		**Genomic regions**					
		**5′UTRs**	**CDSs**	**Introns**	**3′UTRs**	**TEs**	**Intergenic regions**
Mono-	# of P-SSRs	115	26	72,591	598	58,987	135,726
	#/Mb	59.10	0.76	104.31	78.20	49.01	91.61
Di-	# of P-SSRs	60	16	457,58	316	27,997	89,153
	#/Mb	30.83	0.47	54.79	41.32	22.93	62.54
Tri-	# of P-SSRs	178	2,419	362,75	83	6,789	53,607
	#/Mb	91.47	70.38	43.43	10.85	5.35	36.18
Tetra	# of P-SSRs	38	23	134,58	56	12,619	28,975
	#/Mb	19.53	0.67	16.11	7.32	8.58	19.56
Penta-	# of P-SSRs	9	18	328,70	19	3,683	50,814
	#/Mb	4.62	0.52	39.36	2.48	2.94	34.30
Hexa-	# of P-SSRs	5	86	173	2	74	791
	#/Mb	2.57	2.50	0.21	0.13	0.05	0.56
Total	# of P-SSRs	405	2,588	201,125	1074	110,149	359,066
	#/Mb	208.12	75.30	258.21	140.45	88.86	244.75

**Figure 2:**
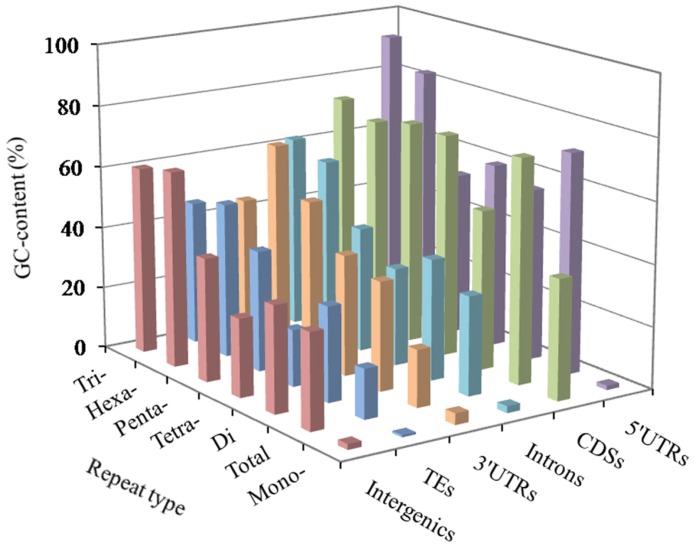
**GC-content of mono- to hexanucleotide P-SSRs in different genomic regions of FMD genome.**

In these genomic regions, the most GC-content occurred in the 5′UTRs (56.73%), next was the pattern: CDSs (53.80%) > 3′UTRs (43.79%) > introns (42.92%) > TEs (41.98%) > intergenic regions (39.42%). The AT- and GC-content of mono- to hexanucleotide P-SSRs were computed in the six genomic regions of the FMD genome, which the results were shown in Figure 2 and Supplementary Table 1. In these different regions of FMD genome, mononucleotide P-SSRs had the least GC-contents. In the 5′UTRs, we can know that the GC-content of di to hexanucleotide repeat types are more than their AT-content; trinucleotide P-SSRs had the most GC-content, next was the pattern: hexa- > tetra- > di- > penta- > mononucleotide P-SSRs. In the CDSs, the GC-contents of tri- to hexanucleotide P-SSRs were relatively high, which were more than their AT-content. In the 3′UTR regions, the GC-content of the mono- to tetranucleotide repeat types were less than their AT-content. In the introns, TEs, and intergenic regions, we can know that tri- and hexanucleotide P-SSRs had the most GC-contents, next was the pattern: penta- > di > tetra- > mononucleotide P-SSRs. There were of similar total GC-contents in the introns and intergenic regions. Therefore, it was inferred that the GC-content of P-SSRs is probably higher in protein-coding regions than that in the rest genomic regions.

### Diversity of most frequent P-SSRs in the six regions of FMD

**Figure 3:**
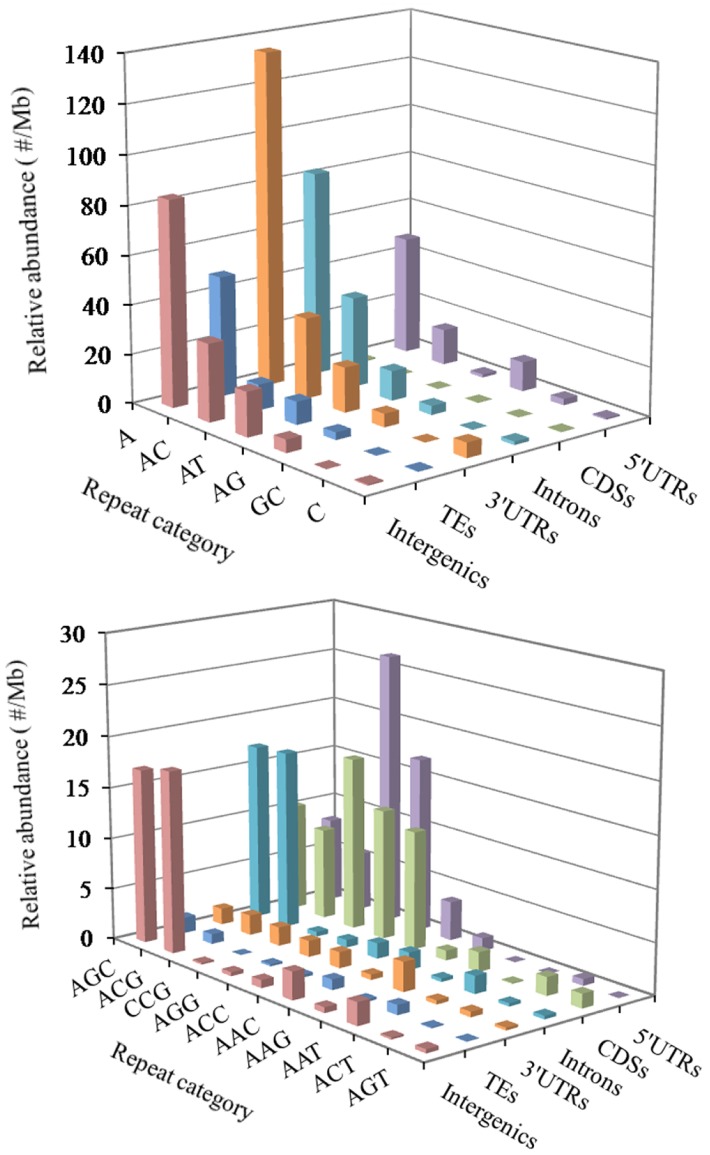
**Distribution of different motifs of mono- to trinucleotide P-SSRs in different genomic regions of FMD genome.**

In the 5′UTR regions, the (A)_n_ was the most abundant repeat unit, the next was the (CCG)_n_, and then were the (AGG)_n_, (AC)_n_, (AG)_n_, (AGC)_n_, (ACG)_n_, and (CCCG)_n_ in order (Figure 3). In the CDS regions, the (CCG)_n_ was the most abundant motifs, next were the (AGG)_n_, (ACC)_n_, (ACG)_n_, and (AGC)_n_, the third were the (AAG)_n_ and (ACT)_n_ (Figure 3). In the intronic regions, the (A)_n_ was the most abundant motif, the next was the (AC)_n_, and then were the (ACG)_n_, (AGC)_n_, (AT)_n_, (AG)_n_ and (AAAT)_n_ in order (Figure 3). In the 3′UTR regions, the (A)_n_ was the most abundant unit, next was the (AC)_n_, and then were the (AT)_n_, (AG)_n_ and (C)_n_ in order (Figure 3). In the TEs, the (A)_n_ was the most common repeat unit, the next were the motif (AC)_n_ and (AT)_n_, and then were the (AG)_n_, (AAAT)_n_, (AAT)_n_, (AAC)_n_, (AGC)_n_, and (AAAC)_n_ in order (Figure 3). In the intergenic regions, the (A)_n_ was the most abundant repeat unit, next was the motif (AC)_n_, and then were the (AT)_n_, (AGC)_n_, (ACG)_n_, (AG)_n_, (AAAT)_n_, (AAC)_n_, (AAT)_n_, and (AAAC)_n_ in order (Figure 3). Therefore, it is inferred that there are the nonrandom distribution of P-SSR motifs in these different genomic regions.

### The analysis of coefficient of variability (CV) of SSRs in different genomic regions of FMD

**Figure 4:**
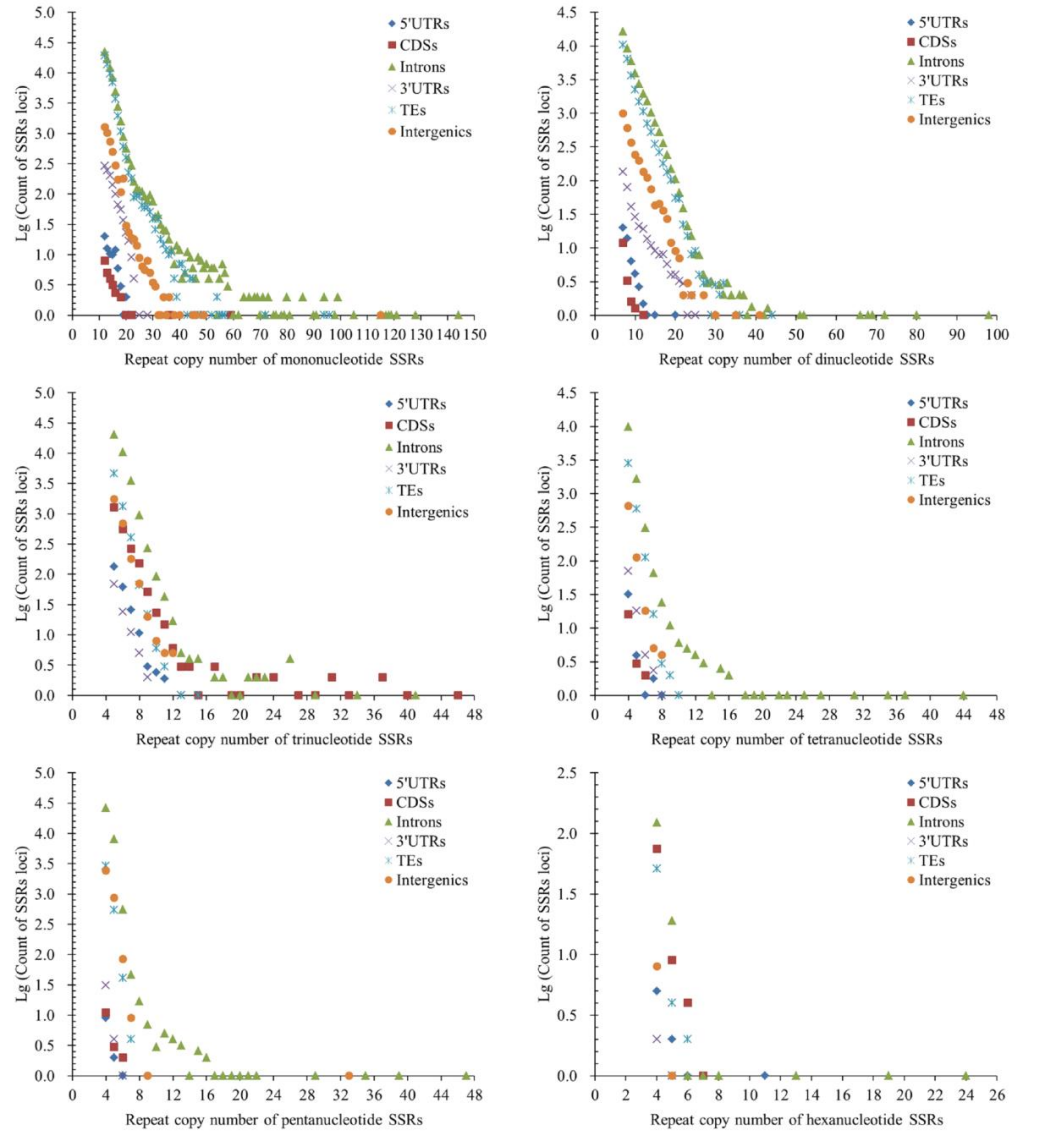
**Comparative analysis of repeat copy number (RCN) of mono- to hexanucleotide P-SSRs in different genomic regions of FMD genome.**

The repeat copy numbers (RCN) of the same P-SSRs had significantly difference in these genomic regions of FMD genome. In the RCN of mono- and dinucleotide P-SSRs, the intron regions and TEs had the most counts of SSRs loci, followed by the pattern: intergenic regions > 3′UTRs > 5′UTRs > CDSs (Figure 4). The RCN of trinucleotide P-SSRs had increased obviously in the CDS regions comparing with the 3′UTR and 5′UTR regions, which had more counts of SSRs loci (Figure 4).

The RCN of trinucleotide SSRs had the fewest numbers of SSRs loci in the 5′UTR and 3′UTR regions (Figure 4). In the RCN of tetra- and pentanucleotide P-SSRs, the intron regions had the most numbers of SSRs loci, followed by the TEs and intergenic regions, the 5′UTR and CDS regions had fewer counts of SSRs loci (Figure 4). In the RCN of hexanucleotide P-SSRs, the numbers of P-SSRs loci had increased obviously in the CDSs regions in comparison to other genomic regions (Figure 4). The results showed that the RCN of different P-SSRs was significantly decreased with increasing of nucleotide repeat units.

**Figure 5:**
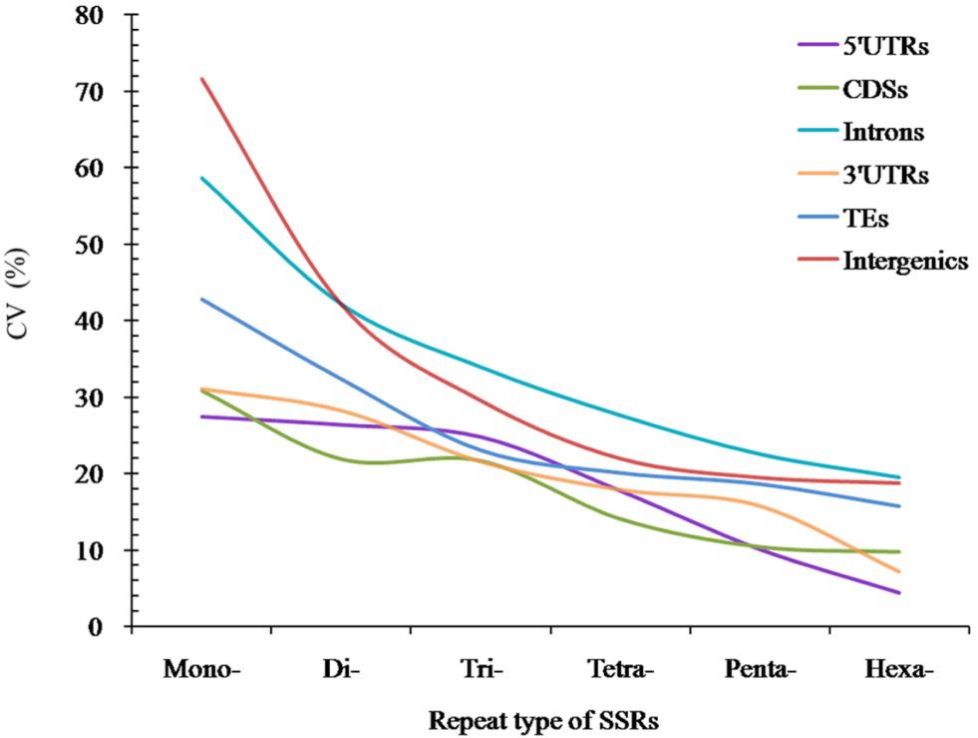
**The CV analysis of RCN of P-SSRs in different genomic regions of FMD genome.**

The analysis of coefficient of variability (CV) of SSRs showed that the RCN variation of different P-SSRs had significantly decreased from mono- to hexanucleotide P-SSRs (Figure 5). In the CDS region, there were of similar CV variation in the di- and trinucleotide P-SSRs (Figure 5). The CV variation of the same P-SSRs had obviously difference in the six regions of FMD genome. The intronic and intergenic regions had relative higher CV variations of the RCN for the same P-SSRs, followed by the TEs, and the rest genomic regions had relative lower CV variations of the RCN (Figure 5).

### Development of SSR markers in the FMD

There were 39,977 tetranucleotide SSRs sequences recognized in the FMD genome. By using the above criteria, we isolated 2,160 SSR loci with a RCN of 10 to 22, and then 150 candidate sequences were suited to exploit tetranucleotide SSR markers for the FMD. We synthesized 58 pairs of primer for PCR amplification, the 58 loci of which displayed a single band of anticipated size after amplification. The forward primers of these 58 loci were labelled with different fluorescent dyes and then used to genotype 36 captive FMD (Sichuan, blood DNA). The primers which were not shown a clear single band of expected size or lacked polymorphism, were not used. Finally, 22 novel tetranucleotide SSRs loci were detected for the FMD.

The DNA sequences of these 22 loci were uploaded to NCBI (GenBank accession numbers KT390284–KT390300; KT844392–KT844396). Based on these 22 tetranucleotide SSRs found in this study, we recognized a total of 83 alleles, the number of which is ranged from 2 to 7 for each locus. The observed and expected heterozygosity at each locus ranged from 0.115 to 0.962 and from 0.111 to 0.776, respectively. The PIC ranged from 0.103 to 0.726 with an average of 0.412. The population genetic analysis will be affected if the loci is not sufficient to meet HWE. 1 out of the novel 22 loci deviated obviously from HWE (*P* < 0.01, Locus name: LS-55-1, Accession number: KT844935, motif=(CATA)_14_, Tm=59 °C, k=7, Ar=7.00), which should be deserted.

### Genetic diversity of captive FMD population in china

The genetic diversity of Sichuan captive FMD population was analysed by using the above 21 SSRs markers. The results presented in the Table 4, 76 alleles in total were recognized in the midst of 36 FMDs. The number of alleles ranged from 2 to 6 for each locus. The mean AR was 3.609, ranged from 1.965 to 6.000 alleles. The mean H_O_ and H_E_ were 0.423 and 0.445, espectively, which ranged from 0.115 to 0.692 and from 0.111 to 0.735, respectively. The average PIC was 0.397 with the range of 0.103 - 0.683 in the population. HWE tests indicated that there was not a deviation of HWE for the 21 markers in the population (*P* > 0.01).

**Table 4:** Characteristics of the novel microsatellite marker system and the genetic diversity of captive FMD population, including locus names, primer sequences, accession number, repeat unit, fluorescent dyes, annealing temperatures (Tm), length (bp), numbers of individuals genotyped (N), numbers of alleles (k), observed heterozygosity (H_O_), expected heterozygosity (H_E_), allelic richness (Ar), Polymorphism Information Contents (PIC), HWE P values (*P*-value).

**Locus name**	**Primer sequences (5′—3′)**	**Accession no.**	**Repeat unit**	**Fluorescent dyes**	**Tm (°C)**	**Range (bp)**	**N**	**k**	**H_O_**	**H_E_**	**Ar**	**PIC**	***P*-value**
LS-2-1	F: GATCGAGTTGCAGGAGTC R: CACCATTCAATTCAGAGAAGC	KT390284	(GCAG)_10_	FAM	57	416-440	36	6	0.385	0.65	6.000	0.593	0.03
LS-6-1	F: CAGGATCTGCTTCTGACATT R: ACCAAATCCAACAAGATCCG	KT390285	(GATG)_8_	HEX	59	420-432	36	3	0.538	0.555	3.000	0.484	0.376
LS-7-1	F: TAATTAGAGGGGTGTAAGCG R: GGACCGAGCAAGTAGTAAC	KT390286	(AGGA)_8_	HEX	57	412-428	36	2	0.154	0.145	2.000	0.132	0.884
LS-8-1	F: TGTTCCTGGGATTCTTGAAG R: CATAATTGCCAAAGTGCTGT	KT390287	(AGAC)_8_	FAM	55	408-432	36	5	0.654	0.675	5.000	0.598	0.311
LS-9-1	F: ATGAATCAACTCAGTCCCTG R: GTGGTTAGGACTCAGCATTT	KT390288	(ATAG)_8_	HEX	59	410-430	36	3	0.192	0.278	2.895	0.255	0.054
LS-12-3	F: GCGGGATCATGAGAATAGGT R: CCACATTCTCAAGTTATCCG	KT390289	(CAGA)_8_	FAM	61	408-432	36	3	0.538	0.679	3.00	0.592	0.097
LS-13-1	F: TTGATCCAGTTCAGCAAAGT R: TTTGCAACTTCAATCCACTG	KT390290	(AGAA)_8_	FAM	61	400-432	36	6	0.615	0.655	6.000	0.582	0.253
LS-14-2	F: GGTCTTTCCTG TCACTCCTC R: GTCGCAGCTAC TAATGCTTT	KT390291	(TGCG)_8_	FAM	57	396-432	36	6	0.692	0.735	5.997	0.683	0.562
LS-16-1	F: AGCCATATTCTCAAACCATTC R: CAGAGAGAGACCAGGAACAAC	KT390292	(AGAC)_8_	HEX	57	406-430	36	4	0.577	0.508	3.990	0.454	0.181
LS-17-1	F: TTAACATGACATATGGGAGAG R: TCCAGCATTTTATCATTATTG	KT390293	(TATG)_8_	FAM	57	295-315	36	4	0.615	0.63	4.000	0.548	0.503
LS-18-1	F: CATCCATTCATCTGTCCCTT R: TCCATCCTCCTATCCAAAC	KT390294	(CCAT)_8_	HEX	57	412-430	36	3	0.577	0.562	2.993	0.472	0.391
LS-24-1	F: TTAAACATATGCCTAAGAGTCC R: GCCCAGCAATTCTACTTCT	KT390295	(TTGG)_7_	HEX	57	404-428	36	4	0.615	0.613	4.000	0.555	0.607
LS-27-1	F: CAGGGTAGCTCTAGATTTGT R: GAACTGGCTACTGACATTCT	KT390296	(ATGG)_7_	HEX	55	368-388	36	4	0.308	0.278	3.995	0.257	0.54
LS-28-1	F: CCTAATTTTCCAGCTTGCAG R: AACTGGTGCATGAGTGTATT	KT390297	(ATCC)_7_	FAM	57	396-428	36	3	0.154	0.147	3.000	0.138	0.883
LS-29-1	F: GGAAACACACATCAGAACTC R: GATTCATGTCACTGTATGGC	KT390298	(TTTA)_7_	HEX	57	308-328	36	3	0.346	0.386	3.000	0.343	0.351
LS-30-1	F: CATCACTGAAGCGACTTAGA R: CTGACTTTCCATTGCTACCT	KT390299	(ATCC)_7_	FAM	57	391-403	36	2	0.231	0.208	1.968	0.183	0.722
LS-31-1	F: GTGCTGTATTAGGCTTCAGA R: ATACACACTCATTCCCATCC	KT390300	(ATGG)_7_	HEX	57	408-428	36	2	0.154	0.208	2.000	0.183	0.274
LS-35-1	F: CCCTCAATTCCCTTCGATAG R: TTTGGAGATGATGGACCTTG	KT844932	(TGGA)_7_	FAM	57	178-190	36	2	0.115	0.111	1.965	0.103	0.94
LS-47-1	F: GCCCAGCAATTCTACTTCTA R: AACATATGCCTAAGAGTCCA	KT844933	(CCAA)_15_	FAM	59	220-244	36	4	0.615	0.571	4.000	0.512	0.319
LS-50-1	F: GGGTTGGTATGGAAAGTTCT R: GAATGGGCTTTTATGATGGC	KT844934	(TCCA)_12_	HEX	61	215-235	36	4	0.5	0.455	3.995	0.397	0.235
LS-56-1	F: GTACAGTACCATGCAGTCTT R: AAGTTATCCATCTCCCAACC	KT844936	(CATA)_12_	HEX	59	270-286	36	3	0.308	0.305	3.000	0.277	0.656

## DISCUSSION

In order to ensure the results surveyed by MSDB were more credible, the SSR software Krait [14] was used to verify the consistency of the results, results of which both softwares were consistent. In this study, we discovered 680,635 P-SSRs from the FMD genome, and extrapolated their abundance and genomic distribution in the 5′UTRs, CDSs, 3′UTRs, TEs, intronic and intergenic regions. The development and verification of SSR markers at a whole-genome scale have been shown high intra-specific polymorphic potential [15]. Subsequently, 22 polymorphic SSRs from the FMD genome were recognized and characterized. Our results indicated that the distributional pattern of SSRs in FMD exhibited widespread similarity with the bovid species and deers. P-SSRs occupied 0.43% of FMD genome sequences, the percentage of which was well consistent with *Bos taurus* (0.48%), *Bubalus bubalis* (0.48%), *B. mutus* (0.46%), *Ovis aries* (0.48%), *Capra hircus* (0.46%), *Pantholops hodgsonii* (0.44%) [10], *Odocoileus virginianus* (0.42%), *Giraffa camelopardalis* (0.45%), whereas, these proportions were inconsistent with *Ailuropoda melanoleuca* (0.64%), *Ursus maritimus* (0.79%) [16], and primates (*Otolemur garnetti*: 0.59%, *Callithrix jacchus*: 0.66%, macaques: 0.83%-0.88%, *Chlorocebus sabaeus*: 0.91%, *Papio anubis*: 0.88%, *Nomascus leucogenys*: 0.73%, *Gorilla gorilla*: 0.94%, *Pongo abelii*: 0.73%, *Pan troglodytes*: 0.77%, *Homo sapiens*: 0.74%) [17, 18]. The above proportions of SSRs were analyzed by using the same parameter setting within the above species. These differences may be due to the specificity of species. Mononucleotide SSRs were more than other nucleotide SSR types in the eukaryotic genome [19]. our results also showed that mononucleotide SSRs were the most abundance, which was consistent with previous reports [18, 20–22]. Previous studies have shown that SSRs were frequently associated with TEs [23]. We have demonstrated that there was a similar association between SSRs and TEs. It has been thought that SSRs are derived from another sequence imported by TE or duplications [24, 25]. Short interspersed repeats (SINEs), long interspersed repeats (LINEs) and other retrotransposons are TE classes able to produce SSRs [26]. (AT)_n_ repeats are frequently associated with this TE family in rice [27]. In the human genome a significant positive association also exists between A-rich SSRs and SINEs [28]. This association has been reported in many organisms, however, a great abundance of TEs can′t always combine with a great abundance of SSRs [29].

It has been demonstrated that the RCN of SSRs extension or shrinking directly affected the relevant gene expression and even caused dozens of diseases. The RCN expansions or contraction of (A)_n_ in the CDS regions inactivated mismatch repair (MMR) genes [30, 31], signal transduction genes [32, 33], transcriptional regulation genes [34], apoptosis genes [35] and caused human cancers [36]. It has been reported that trinucleotide SSRs were linked to multiple genetic disorders [37]. SSRs are unstable, the RCN of which can grow from one generation to the next [38]. The expansion of RCN in the CDSs or in the regulatory regions, which may change the gene expression, and even are linked with illness phenotype [39]. In genes of human, the RCN expansion of trinucleotide SSRs are linked with several neurological diseases, such as fragile X syndrome [40], Huntington′s disease and several forms of ataxia [41], and myotonic dystrophy [42]. The RCN expansion of trinucleotide SSRs is usually deemed to be own to DNA replication slippage and unequal recombination in incremental cell [43]. The RCN expansions of (CGG)_n_ in 5′UTRs of the fragile X mental retardation-1 (FMR1) gene were n ≥ 200, which resulted in human mental retardation [44], whereas the (CGG)_40–200_ were also related to cognitive/psychosocial impairment [45], fragile-X-like phenotypes, and woman ovarian dysfunction [46, 47]. The RCN expansions of (CAG)_n_ in 5′UTRs were ranged from 55 to 78 that caused human spinocerebellar ataxia (SCA)12 disease [48]. The RCN expansions of (CTG)_n_ in 3′UTRs led to dystrophia myotonic 1 [49] and SCA8 disease [50]. The RCN expansions of (CA)_n_ in the intron regions could enhance the gene transcription of epidermal growth factor receptor and involve in breast carcinogenesis [51]. The RCN expansions of (GAA)_n_ in the introns inhibited Friedreich′s ataxia (FRDA) gene expression or interfered mRNA formation and led to FRDA disease [52, 53]. The RCN expansions of (ATTCT)_n_ situated in intron 9 of the SCA10 gene which caused SCA10 disease [54]. The RCN of (CAG)_n_ in the human CDSs was expanded for 6 to 35 that performed their normal function, whereas, the (CAG)_36-120_ are translated into lengthened (Gln)_12-40_ tracts within the relevant proteins that resulted in Huntington′s disease (HD) [55]. Extended (Gln)_n_ tracts were poisonous to neurons and peripheral cells alike [56]. It will become the predominant toxic moiety if (Gln)_n_ length is more than regular repeat number [55]. Generally, the numbers of SSRs decreased as the increase of repeat unit and the RCN. This is nearly consistent with that in eukaryotes [5, 57] and prokaryotes [58]. This may be explained by the fact that SSRs with a greater repeat copy number may be more instable due to the increased probability of slippage [26].

SSR distributions are nonrandom and strongly biased in different genomic regions. The counts of mono- and dinucleotide SSR units exceeded other nucleotide SSR motifs in all fungal genomes. In the FMD genome, the most SSR abundance was found in intronic and intergenic regions, followed by the pattern: 3′UTRs > 5′UTRs > TEs > CDSs. The two genomic regions, intronic and intergenic regions, reveal a more similar relative abundance of SSRs in the FMD genome. In the primates, trinucleotide SSRs show approximately double greater abundance in the 5′UTRs than in the CDSs, whereas the latter had much more common trinucleotide P-SSRs than in the intron, 3′UTRs, and TEs [17]. This pattern may be associate with frequencies of amino acids encoded by rich SSRs in the corresponding proteins. It has been verified that SSRs in CDS regions were less abundant than those in intergenic regions [59]. The SSR frequency showed slight difference from the first to the last exon in mammalian species [60]. Tri- and hexanucleotide SSRs predominated in CDS regions of FMD, whereas, tetra- and pentanucleotide SSRs were less abundant in this regions. These trifold SSRs are chosen to avoid possible frameshift mutation. Non-random distributions of trifold SSRs and amino acid repeats have been found in different functional proteins [5, 61, 62], suggesting that these repeats are subject to natural selection [63]. SSRs situated in exonic regions may play a role in gene transcription, regulation, mRNA splicing, and gene silencing [64]. Polymorphic SSRs in CDSs, 5′UTRs or 3′UTRs could change the gene expression and/or protein structure, which may have a role to play in the mechanisms of adaptation, survival and evolution of species. The RCN expansions or contraction of (A)_n_ in the CDS regions of MMR genes could give rise to frameshift mutation in MMR-deficient cells [30]. Furthermore, the difference of SSR abundance between 5′UTRs and 3′UTRs could play the regulatory role of protein translation and/or mRNA steadiness [65]. We well known that SSRs in 3′UTRs can be involved in transcription slides by extension and result in phenotypic disorders [49, 64]. SSR evolution in CDS regions is similar to those of SSRs in 5′UTR and 3′UTR regions, but is not similar to those of SSRs in intron and intergenic regions. The SSRs in CDS regions reveal a higher mutation rate than that in non-repeating sequence regions. SSR variations in the CDS and UTR regions could resulted in frameshift mutation, gene expression silencing, loss of protein function, and even multiple diseases.

It has been reported that the abundance of SSRs in protein-coding regions shows an incline to some specific motifs. The (A)_n_ was more common than the (C)_n_ in the CDS regions of FMD, this is consistent with primates [17, 66]. In the CDSs of FMD, the (CCG)_n_ were the most predominant triplet repeat units, the next were the (AGG)_n_, (ACC)_n_, (ACG)_n_, and (AGC)_n_. In 10 primates, the (AGC)_n_ and (AGG)_n_ were the most abundant repeat units, the next were the motif (CCG)_n_ and (ACG)_n_ in the coding regions [17, 20]. The (CCG)_n_ and (ACG)_n_ translating for (Ala)_n_, (Gly)_n_, (Arg)_n_, and (Pro)_n_ were relatively abundant in primate genes [67]. The (AAT)_n_ and (AAC)_n_ are rarely present in the coding regions of FMD. The (AAT)_n_ motifs can also act as a stop codon, which may explain their lower occurrences in CDSs. The (CG)_n_ and (CCG)_n_ repeats were poorly represented in the 3′UTRs, TEs, intronic and intergenic regions of FMD, this corresponded with the previous report in the primates [17]. The (CCG)_n_ motifs were the most abundant repeats in 5′UTRs and CDSs of FMD. In ten primates, the (CCG)_n_ units were the most common motifs in 5′UTRs and were the second most frequent motif in the CDSs [17]. The RCN expansion or contraction of (CCG)_n_ units may influence gene function. 5′UTRs and CDSs of genes contain the regulatory factors and CpG islands, it is likely that these motifs might regulate gene expression [68]. The (AAC)_n_ and (AAT)_n_ repeats relatively predominated in introns, TEs, and intergenic regions of these primates, which both outnumbered that of other trinucleotide repeats [17, 20]. These results suggested that amino acid repeats could happen in the coding sequences. It has reported that (Asn)_n_ motif existed in mammalian proteins [69]. It is widely assumed that repeat copy number of amino acids are relatively within the normal range and may not affect the stability, structure, and function of the protein.

## MATERIALS AND METHODS

### Genome sequences and genomic regions

We have sequenced the whole genome of FMD, completed the genomic assembly and annotation, and performed preliminary bioinformatic analyses. With the K-mer method supplied in GCE-1.0.0 [70], the genome size of FMD was estimated to be 2.72 Gb, which was similar to that of sheep (*Ovis aries*, 2.61 Gb) [71], of goat (*Capra hircus*, 2.66 Gb) [72], and Tibetan antelope (*Pantholops hodgsonii*, 2.75Gb) [73]. According to the calculated genome size, the clean data provided 130× average coverage. Collectively, the *de novo* genome assembly contained 2,143,175,501 contigs with a N50 contig size of 22.6 kb. Those contigs were then assembled to yield 1,114,025 scaffolds whose N50 size was 2.85Mb. The sequences of the gene models, 5′UTRs, CDSs, introns, 3′UTRs, TEs, and intergenic regions were generated according to the positions in the genome annotations. The intergenic regions referred to the interval sequences between gene and gene that were not included the introns, CDSs, UTRs, and other related sequences.

### SSRs identification and investigation

As FMD had very large genomes, relatively systemic search criteria [74] were adopted in the study. In this study, repeats with unit patterns being circular permutations and/or reverse complements of each other were grouped together as one type for statistical analysis [75, 76]. For tetra- and hexanucleotide repeats, relatively systemic combination criteria were applied [10] in the process of filtration. To facilitate the comparison among different repeat categories or motifs, the term of relative abundance was used in the study (see the literatures: [74, 77]). SSRs were identified and scanned for 1-6 bp using the software MSDB (Microsatellite Search and Building Database) [74]. These total numbers have been normalized as relative abundance to allow comparison in the different genomic regions. In the four DNA bases, percentage of guanine (G) plus cytosine (C) was called GC-content in the analyzed sequence [17].

### Development of polymorphism SSR markers

The flanking regions of microsatellites (200 bp either side) were extracted from the FMD genome in order to design the primer sets for the SSR loci identified. These extracted sequences were further manually scanned and filtered according to the criteria of SSRs identification as follows: (1) SSRs should be tetranucleotide repeats; (2) the number of repeats should be in the range of 7–15; (3) other criteria were used (see the previous report [78]). We used Primer 3 to design the primers to amplify the selected sequences [79]. The lengths of the primers designed in the present study were between 18 and 23 bp, with an expected product size between 190 and 450 bp. Amplification of these primers were tested in the FMD under the standard PCR conditions according to the change of annealing temperature of primer sequence. During optimization, we tested whether amplification was improved by the addition or decrease of MgCl_2_, or by a higher or lower annealing temperature. After optimization, the primers with single band of expected size in the amplification were selected to label with one of two fluorescent dyes (FAM, HEX) in the forward primers for fragment analysis on Applied Biosystems 3100 Genetic Analyzers. The blood DNA from 36 captive FMD was used to evaluate the ability of the primer pairs to amplify polymorphic bands. PCR amplifications were carried out in 25 μL reaction mixtures, and amplifications were performed using the standard PCR procedure [78]. For genotyping of SSR loci, the PCR amplification products were separated by capillary electrophoresis using a denaturing acrylamide gel matrix on an ABI PRISM 377 Genetic Analyser (Applied Biosystems) using GeneScan Tarmara 350 internal size standard (ABI). Alleles were detected using the GeneScan⁄Genotyper software package of Applied Biosystems. If the SSR markers had stutter peaks, they would be eliminated in this step. The ‘multi-tube procedure’ [80] was used to test the tendency for genotyping errors in these SSR loci.

### Statistical analysis

Micro-Checker software [81] was used to estimate the presence of genotyping errors, such as null alleles, large allele dropout, or stuttering in the data set. The number of alleles (A), observed heterozygosity (H_O_), expected heterozygosity (H_E_), polymorphic information content (PIC), and the paternity test were calculated by using the software of CERVUS 3.0 [82]. Tests for deviations from the Hardy–Weinberg equilibrium (HWE) and linkage disequilibrium (LD) were performed using GENEPOP 3.4 [83]. Allelic richness (Ar) was calculated by using the FSTAT 2.9.3 program package [84]. Individual identification was analysed by CERVUS 3.0 [82]. In order to analyze the variation of repeat copy numbers (RCN) of different repeat type SSRs in the different genomic regions, we introduced the coefficient of variability (CV), which the calculation formula was as follow: $\text{CV}=\text{S}/\bar{x}\times 100\%$. Where S was the standard deviation of the RCN of one SSR, $\bar{x}$ was the average of the RCN. The variation of RCN of two or more SSRs were comparative analyzed by the CV, which could eliminate the effect of different unit and mean, and be able to really reflect variation level of RCN of different SSRs.

### Availability of supporting data

The DNA sequences have been uploaded into the NCBI (GenBank accession numbers KT390284–KT390300; KT844392–KT844396).

## Supplementary Material

Supplementary Table 1
