# Supplementary material for: Distribution patterns of microsatellites and development of its marker in different genomic regions of forest musk deer genome based on high throughput sequencing
Source: Aging (Albany NY). 2020 Mar 10;12(5):4445–62. doi: 10.18632/aging.102895 (PMC7093171; doi:10.18632/aging.102895)
Supplement: Supplementary Table 1 [file aging-12-102895-s001..pdf]

## SUPPLEMENTARY TABLE

**Supplementary Table 1. The AT-content (%) of mono- to hexanucleotide P-SSRs in the different genomic regions of the forest musk deer genome.**

| Type | 5′UTRs | CDSs | Introns | 3′UTRs | TEs | Intergenics |
|---|---|---|---|---|---|---|
| Mono- | 98.57 | 61.05 | 97.80 | 96.29 | 99.29 | 98.16 |
| Di- | 44.31 | 48.17 | 60.84 | 64.67 | 69.23 | 65.51 |
| Tri- | 7.70 | 26.55 | 37.55 | 55.48 | 53.58 | 39.33 |
| Tetra- | 40.16 | 27.69 | 68.31 | 60.85 | 81.61 | 74.58 |
| Penta- | 47.32 | 27.61 | 59.52 | 47.73 | 60.87 | 60.04 |
| Hexa- | 16.67 | 30.53 | 41.35 | 33.33 | 49.86 | 36.49 |
| Total | 28.35 | 27.23 | 67.92 | 81.51 | 83.95 | 69.02 |
